# Epidural injection of dexamethasone palmitate vs. betamethasone for lumbar radiculopathy, study protocol for a multi-center non-inferiority randomized double-blind controlled trial

**DOI:** 10.3389/fphar.2026.1801501

**Published:** 2026-05-18

**Authors:** Peng Huang, Liqun Huang, Junpeng Yuan, Jun Guo, Chunting Lu, Xia Zhang, Mingming Liu, Yang Wang, Liyong Zhang, Jiakai Li, Xiangnan Li, Hongjin Zhang, Ke Wang, Xin Jin, Xiaohong Jin

**Affiliations:** 1 Department of Pain, The First Affiliated Hospital of Soochow University, Suzhou, China; 2 Department of Pain Medicine, The Fourth Affiliated Hospital of Soochow University, Suzhou, China; 3 Department of Pain, Suzhou Wuzhong People’s Hospital, Suzhou, China; 4 Department of Anesthesiology, Suzhou Wuzhong People’s Hospital, Suzhou, China; 5 Department of Pain, The Second People’s Hospital of Lianyungang, Lianyungang, China; 6 Department of Pharmacy, Yangzhou Hospital of TCM, Yangzhou, China; 7 Department of Orthopedics, Yangzhou Hospital of TCM, YangZhou, China; 8 Department of Pain, Lianyungang Municipal Oriental Hospital, Lianyungang, China; 9 Department of Pain, Yancheng Third People’s Hospital, Yancheng, China

**Keywords:** betamethasone (BTM), dexamethasone palmitate (PubChem CID:63,044), epidural steroid injections, lumbar disc hemiation, lumbar radiculopathy

## Abstract

**Introduction:**

Lumbar disc herniation is commonly treated with epidural steroid injections to relieve radicular pain. Dexamethasone palmitate, a newer lipophilic formulation, is theoretically safer and provides longer-lasting effects compared to traditional corticosteroids like betamethasone. However, direct clinical evidence comparing their efficacy and safety is still limited.

**Methods:**

This multicenter, randomized, double-blind, non-inferiority trial will enroll 228 patients with confirmed lumbar disc herniation and radicular pain. Participants will be randomly assigned (1:1) to receive an image-guided epidural injection of either DEP or compound betamethasone via interlaminar or transforaminal approach. Follow-up assessments will occur at 1 day, 1 week, 2 weeks, 4 weeks, 8 weeks, and 12 weeks post-injection. The primary outcome is leg pain intensity at 4 weeks, measured by the numeric rating scale (NRS). Secondary outcomes include NRS at other time points, functional disability (ODI and RMDQ), mental health (GAD-7 and PHQ-9), morning serum cortisol, patient satisfaction, and adverse events. A second injection may be administered at 4 weeks based on clinical response.

**Expected outcomes:**

This study will provide the first high-quality evidence on the efficacy and safety of DEP compared with standard particulate corticosteroids for lumbar radiculopathy. Findings are expected to inform clinical decision-making and optimize steroid selection for epidural therapy.

**Clinical Trial registration:**

[https://www.chictr.org.cn], identifier [ChiCTR2400093970].

## Introduction

Lumbar disc herniation is a common cause of radicular pain and disability in adults, resulting from nerve root compression by displaced disc material (Ropper and Zafonte, 2015; Deyo and Mirza, 2016). Epidural steroid injections (ESIs) are widely used and recommended by the North American Spine Society (NASS) guidelines as a cost-effective option that provides significant pain relief in patients with lumbar radiculopathy (McLain et al., 2005; Liu et al., 2023; Kreiner et al., 2014).

Steroids used in ESIs are categorized as particulate and non-particulate types, and differ in their pharmacokinetics, duration of action, and potential side effect profiles. Particulate steroids, such as triamcinolone and compounded betamethasone preparations, have a larger molecular size and remain at the injection site longer due to their delayed absorption (Benzon et al., 2007), a property that theoretically allows for a prolonged anti-inflammatory effect, and may result in more sustained pain relief (Cole and Schumacher, 2005). However, recent case reports have documented instances of paraplegia in patients following lumbosacral nerve root blocks that utilized the particulate steroids betamethasone or methylprednisolone acetate (Aprill and Melfi, 2004; Houten and Errico, 2002). The use of particulate corticosteroids for epidural injections has also been cautioned against by the FDA in 2011 and 2014, stating that “injections into the epidural space may result in rare but serious adverse events, including loss of vision, stroke, paralysis, and death.” Although particulate steroids are associated with rare but serious complications, including spinal cord infarction, they continue to be used in clinical practice, particularly for interlaminar epidural injections or when a longer-lasting anti-inflammatory effect is clinically desirable.

Compared to particulate steroids, non-particulate types (e.g., dexamethasone) are regarded as a safer option for injection into high-risk areas, such as the epidural space and around blood vessels, as they are unable to induce ischemic events due to their small crystalline structure.

Previous studies comparing particulate and non-particulate steroids have suggested that particulate formulations may provide superior analgesic effects and greater short-term improvement in pain outcomes (Bensler et al., 2017).

Dexamethasone palmitate (DEP) a lipophilic prodrug of dexamethasone, has been utilized in clinical practice for the treatment of various conditions, including rheumatoid arthritis and post-transplant hemophagocytic lymph histiocytosis (Lorscheider et al., 2019; Ono et al., 2023). However, no published studies have yet evaluated its efficacy in epidural injection for lumbar disc herniation. Previous studies have suggested that particulate corticosteroids may provide superior and more sustained analgesic effects compared with non-particulate formulations in epidural steroid injection therapy. In this context, it remains unclear whether dexamethasone palmitate, as a novel corticosteroid formulation with distinct pharmacokinetic properties, can achieve comparable clinical efficacy to particulate steroids in the treatment of lumbar radiculopathy. Therefore, a non-inferiority design will be adopted to determine whether dexamethasone palmitate can provide clinically acceptable and comparable outcomes to a commonly used particulate corticosteroid regimen, while potentially offering additional pharmacological or safety advantages.

## Methods and analysis

### Study design

This is a two-arm multi-center, non-inferiority, randomized double-blind controlled trial. Patients with clinically confirmed lumbar disk herniation and radicular pain will be randomly assigned to one of two treatment arms: image guided epidural injection of DEP or combined betamethasone through an interlaminar or transforaminal approach. Follow up will be conducted at 1 day, 1 week, 2 weeks, 4 weeks, 8 weeks, and 12 weeks post-treatment. Pain intensity will be evaluated at all follow up time points, functional outcomes and mental health will be evaluated at 4 weeks, 8 weeks, and 12 weeks follow-up time points. Safety outcomes, including side effects and adverse events will be assessed at 1 day and 4 weeks after treatment. For patients with a pre-existing diagnosis of diabetes, fasting blood glucose levels will be monitored daily for seven consecutive days postoperatively to evaluate the impact of steroids on glycemic control. A repeat injection will be administered based on the patient’s response at 4 weeks of follow-up.

Approval for this study was obtained from the Research Ethics Committee of all the center (approval reference number: 2024-457, 2025-251001, 2024K095, KY-2024-003-02, 2025-06, 2024-41, 2025-002). The trial was registered in the Chinese Clinical Trial Registry (Trial number: ChiCTR2400093970, https://www.chictr.org.cn, Chinese Clinical Trial Registry, 2024-12-15) and the trial protocol followed the ‘Standard Protocol Items: Recommendations (SPIRIT) guideline (Schulz et al., 2010). Patient consent was obtained after enrollment in accordance with the Declaration of Helsinki.

### Study setting

**FIGURE 1:**
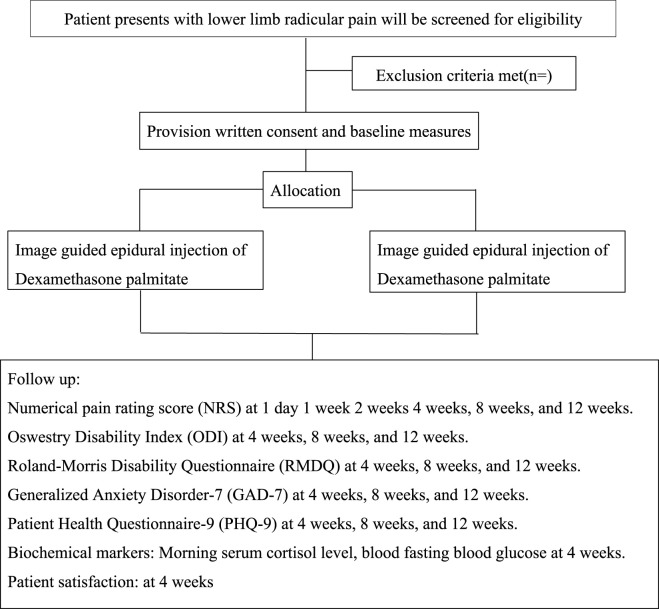
Participant flow chart.

This study will be conducted across seven medical institutions located in Jiangsu Province, China. The participating centers include pain management or orthopedic surgery department in each hospital that have expertise in managing lumbar disk herniation. All have the capacity to conduct clinical trials with appropriate infrastructure and resources, including advanced imaging and pain management facilities, allowing for precise diagnosis and effective treatment delivery (Figure 1).

### Participant eligibility and trial recruitment

Participants will be recruited primarily from outpatient clinics across the seven participating medical institutions and screened for eligibility based on the inclusion and exclusion criteria outlined below:

### Inclusion criteria

1. Age ≥18 years.
2. Unilateral buttock or lower limb radicular pain, with or without low back pain.
3. Leg or buttock pain greater than low back pain, with a Numerical Rating Scale (NRS) score ≥4.
4. Magnetic Resonance Imaging (MRI) or Computed Tomography (CT) of the lumbar spine within the past 6 months, confirming the presence of one level lumbar disc herniation with nerve root compression, with clinical symptoms and signs consistent with imaging findings.

### Exclusion criteria

1. Individuals with known allergies to the study medication.
2. A history of psychiatric disorders.
3. Inability to comprehend the study details, participate in scale assessments, or comply with follow-up visits.
4. Presence of comorbid conditions that could affect the assessment of treatment efficacy, specifically conditions that could cause leg pain (e.g., Parkinson’s disease, diabetic peripheral neuropathy, lower limb thrombosis).
5. Severe cardiovascular diseases that impair daily activities or affect functional assessment scores.
6. Unstable spondylolisthesis (vertebral displacement >3 mm or endplate angle change >15° on dynamic lumbar spine radiographs), or Grade 3 and above spondylolisthesis with instability.
7. Severe osteoporosis with vertebral compression fractures in the segment intended for injection.
8. Malignant tumors with spinal metastasis.
9. Pregnant or breastfeeding women, women planning pregnancy soon, or women whose partners are planning pregnancy.
10. Other contraindications to injection treatments, such as current infection at the puncture site, severe coagulopathy and resulting complications (e.g., gingival bleeding)
11. Recent (within 3 months) history of spinal intrathecal injections.
12. Upcoming surgical plans within the next 3 months.
13. Platelet count below 50 × 10^9^/L or concurrent use of anticoagulant or antiplatelet medications.
14. Presence of the following conditions: posterior subcapsular cataract, glaucoma, herpes simplex keratitis, poorly controlled diabetes (fasting blood glucose ≥7 mmol/L), acute myocardial infarction, peptic ulcer, tuberculosis, infections without effective antibiotic treatment, systemic fungal infections, electrolyte imbalances, recent abdominal surgeries, or thrombosis.

### Study sample size

Assuming there is no difference in the mean NRS score between groups at 4 weeks after treatment, with a standard deviation of 2.2, a two-sided α of 0.05 (equivalent to a one-sided α of 0.025), and a power (1–β) of 0.90, and setting a non-inferiority margin of 1 (less than the minimal clinically important difference of 1.5), a 1:1 allocation ratio between the experimental and control groups was used. Based on PASS software calculations, 102 participants per group were required. After accounting for an estimated 10% loss to follow-up and refusal rate, the final required sample size was determined to be 114 participants per group (Feeley et al., 2017).

A non-inferiority margin of one point on the NRS was selected based on the established minimal clinically important difference (MCID) for radicular pain. This margin was chosen to balance statistical rigor with clinical relevance, allowing detection of whether dexamethasone palmitate maintains acceptable analgesic efficacy compared with standard corticosteroid therapy. From a clinical perspective, demonstrating non-inferiority would support the use of dexamethasone palmitate as a viable alternative treatment option, particularly if it offers additional pharmacological advantages such as prolonged tissue retention or improved safety characteristics.

### Randomization and blinding

Randomization will be performed using a computer-generated random sequence with a 1:1 allocation ratio. Block randomization stratified by study center, duration of symptoms (disease course), and Pfirrmann scale will be applied to ensure balanced distribution of key prognostic factors across treatment groups. The random sequence will be generated by an independent statistician who is not involved in patient recruitment, treatment administration, or outcome assessment.

Allocation concealment will be ensured using a centralized, password-protected randomization system. After enrollment, patients will be assigned to treatment groups by a designated research coordinator at each center, who will not participate in outcome assessment or data analysis.

This study adopts a double-blind design. Participants, treating physicians, outcome assessors and statistician will remain blinded to group allocation.

A dedicated research staff member at each center is responsible for preparing the study medication according to the randomization assignment. The allocated drug is drawn into identical, pre-labelled syringes that are indistinguishable in appearance. After preparation, each syringe is completely covered with an opaque protective sheath to prevent visualization of the solution characteristics.

To further maintain blinding during administration, an opaque injection needle is used so that the appearance of the injectate cannot be observed at any point during the procedure. The syringe and needle remain connected throughout the injection process and are withdrawn as a single unit without disconnection. Immediately after injection, all used materials are handed over to designated unblinded personnel for disposal.

Blinding will be maintained throughout the entire follow-up period. In case of a serious adverse event requiring emergency unblinding, the allocation code may be disclosed by the independent coordinator after appropriate authorization, and all unblinding events will be documented.

### Measurement of clinical outcomes

Baseline demographic characteristics, including gender, age, education level, occupation, smoking history, allergy history, family medical history, history of diabetes, and current use of analgesics, will be assessed, in addition to patient measurements such as height, weight, the level of lumbar disc herniation, and the severity of nerve compression measured by Pfirmann grading system base on lumbar MRI (Grade I: Herniated disc in contact with the nerve root without displacement; Grade II: Herniated disc in contact with the nerve root, no nerve root displacement; Grade III: Herniated disc in contact with the nerve root, causing posterior displacement of the nerve root; Grade IV: Herniated disc in contact with the nerve root, compressing it against the posterior wall of the spinal canal) (Majeed et al., 2016). Laboratory parameters, including fasting blood glucose and morning serum cortisol levels will also be measured. Pain intensity will be evaluated using the NRS for both low back pain and leg pain. The Oswestry Disability Index (ODI) and Roland-Morris Disability Questionnaire (RMDQ) will be used to assess functional disability. Generalized Anxiety Disorder 7 (GAD-7) and Patient Health Questionnaire 9 (PHQ-9) were used to assess anxiety and depression.

Follow-up assessments will be conducted at post-injection day 1, week 1, week 2, week 4, week 8, and week 12 (Table 1).

**TABLE 1:** Schedule of enrolment, interventions, and assessments.

Timepoint	Enrolment	Baseline	Allocation	Follow up					
	​	​	0	*Day1*	*Week* *1*	*Week* *2*	*Week* *4*	*Week* *8*	*Week* *12*
Enrolment									
Eligibility screen	X	​	​	​	​	​	​	​	​
Informed consent	X	​	​	​	​	​	​	​	​
Allocation	​	​	X	​	​	​	​	​	​
Interventions									
Dexamethasoe palmitate	​	​	X	​	​	​	​	​	​
Combined betamethasone	​	​	X	​	​	​	​	​	​
Assessmens									
*Pain intensity (NRS score)*	​	X	​	X	X	X	X	X	X
*ODI score*	​	X	​	​	​	​	X	X	X
*RMDQ score*	​	X	​	​	​	​	X	X	X
*PHQ-9*	​	X	​	​	​	​	X	X	X
*GAD-7*	​	X	​	​	​	​	X	X	X
*Serum cortisol* *Levels blood glucose*	​	X	​	​	​	​	X	​	​
*Satisfaction*	​	​	​	​	​	​	X	​	​
*Adverse event*	​	​	​	X	X	X	X	X	X

The primary outcome of this study is leg pain measured by NRS score which is measured on a scale of 0–10, with 0 indicating “no pain” and 10 indicating “the worst pain imaginable” at 4 weeks after procedure.

The secondary outcomes are:

Pain: NRS scores at 1 week, 2 weeks, 8 weeks, and 12 weeks post-injection.

Functional disability: ODI and RMDQ at 4 weeks, 8 weeks, and 12 weeks post-injection.

Mental health assessment: GAD-7 and PHQ-9 at 4 weeks, 8 weeks, and 12 weeks post-injection.

Biochemical markers: Morning serum cortisol levels at 4 weeks post-injection. For participants with a pre-existing diagnosis of diabetes, fasting blood glucose will be monitored daily for seven consecutive days post-injection.

Patient satisfaction: Patient satisfaction will be evaluated at 4 weeks post-injection using a structured questionnaire. Satisfaction will be rated on a five-point Likert scale: very dissatisfied, dissatisfied, neutral, satisfied, and very satisfied. In addition, patients will provide a global impression of treatment effect, categorized as: marked improvement, moderate improvement, slight improvement, no change, mild deterioration, moderate deterioration, or severe deterioration.

Throughout the follow-up period, patients will be allowed to continue their pre-existing medication regimens. Concomitant medication use, including nonsteroidal anti-inflammatory drugs (NSAIDs), tricyclic antidepressants (TCAs), and gabapentinoids, will be recorded in detail at each visit, including type, dose, frequency, and duration.

Incidence of adverse events defined as any unintended and harmful outcome that occurs during or after the procedure, which may be directly or indirectly associated with the intervention.

### Intervention

Patients will receive at least one epidural injection of compound betamethasone, a corticosteroid preparation that includes 2 mg of betamethasone sodium phosphate and 5 mg of betamethasone dipropionate or dexamethasone palmitate 4 mg (contains 2.5 mg dexamethasone). The choice of compound betamethasone as a comparator in this study is based on its established efficacy and widespread use in managing inflammation and pain associated with lumbar disk herniation (Lee et al., 2022).

### Intervention description

Certified pain management physicians and orthopedic surgeons with expertise in administering epidural glucocorticoid injections will perform all procedures. The physician chose the approach (transforaminal or interlaminar) according to their usual practice. Physicians in this study were trained by the study investigators to administer the injections in a standardized manner with the use of image guidance (e.g., CT, digital subtraction angiography, or C-arm).

The injection level will be determined by MRI and the patient’s symptoms. For interlaminar injection, the level of injection will select at the spinal level of lumbar disk herniation. For the transforaminal approach, the nerve root chosen is based on prior nerve root compression identified on MRI or CT.

Patients are placed in the prone position for the procedure while a needle is inserted through the interlaminar or transforaminal route, targeting the nerve root. Upon reaching the nerve root, a sensation of nerve root paresthesia will be elicited. A contrast agent is injected alone initially to confirm the appropriate placement of the needle and ensure that the contrast reaches the epidural space.

Following this, a mixture of 0.2%–0.5% lidocaine and either DEP or betamethasone, along with the contrast agent, will be slowly injected for a total volume of 5 mL to ensure proper distribution.

For the final step, fluoroscopic imaging is performed to confirm that the injected solution properly entered the epidural space, ensuring the accuracy of the injection before the procedure is completed.

### Repeat injection

At the 4-week follow-up, patients who experience ≥50% reduction in leg pain from baseline may be considered for a repeat injection. The medication and route of administration for the repeat injection will be the same as those used in the initial treatment. Patients may also choose to receive the alternate study medication for the repeat injection. Decisions regarding repeat injections will be made collaboratively between the physician and the patient and documented in the case report form. Each patient will receive one injection initially, with a maximum of two injections allowed during the study period.

### Criteria for trial discontinuation

Participation in the trial may be discontinued under the following conditions:

1. Occurrence of any serious adverse event related to the study intervention;
2. Significant worsening of symptoms, including increased pain intensity or new or progressive neurological deficits (e.g., motor weakness, sensory loss);
3. Development of procedure-related complications (e.g., infection, bleeding, or suspected vascular injury);
4. Any unexpected clinical condition that, in the judgment of the primary investigator, makes continued participation unsafe;
5. Withdrawal of consent by the participant at any time.

All discontinuation events will be documented, including the reason and timing, to ensure transparency in data analysis.

### Strategies to improve adherence to interventions

The use of non-steroidal anti-inflammatory drugs (NSAIDs) or tramadol as rescue medication is permitted during the study period. Allowed medications are limited to ibuprofen (maximum 1,200 mg/day) or naproxen (maximum 1,000 mg/day) or tramadol at a maximum 400 mg/day administered orally in divided doses as needed for pain control. Patients are instructed to use rescue medication at the lowest effective dose and to maintain consistent usage patterns, when possible, throughout the follow-up period.

The type, dose, and frequency of NSAID use will be recorded at each follow-up visit and included in the statistical analysis to assess potential confounding effects.

Additional invasive interventions targeting lumbar disc herniation, such as nerve blocks or spinal cord stimulation, are not permitted during the study period, as they may directly influence pain and functional outcomes.

All clinical staff involved in administering the intervention will receive comprehensive training on the protocol. Each center is also provided with easy access to detailed protocol documentation, including visual aids and quick-reference guides that summarize key points of the protocol. Periodic audits will be conducted at each center to review adherence to the protocol.

### Statistical analyses

Descriptive statistics are used to summarize baseline characteristics (e.g., demographic information, clinical variables). Continuous variables are presented as mean with standard deviation or median with interquartile range, depending on the distribution of the data. Categorical variables are presented as frequencies and percentages. Statistical analysis is performed by investigators blinded from the allocation group, according to the per protocol and intention-to-treat principles.

### Primary outcome analysis

The primary outcome (NRS score at 4 weeks) will be analyzed using a linear mixed-effects model, including treatment group, time, and their interaction as fixed effects, with study center as a random effect, baseline NRS score will be adjusted as a covariate.

Non-inferiority will be assessed based on the between-group difference in mean NRS score at 4 weeks post-treatment, with a pre-specified non-inferiority margin of one point. Non-inferiority will be concluded if the upper bound of the two-sided 95% confidence interval for the between-group difference does not exceed the non-inferiority margin. Both intention-to-treat (ITT) and per-protocol (PP) analyses will be performed to ensure robustness of the results.

### Secondary outcome analysis

The secondary outcomes of this study are leg pain scores at multiple follow-up time points, functional disability (ODI and RMDQ), mental health assessment (GAD-7 and PHQ-9), and biochemical markers. For leg pain, the NRS scores for leg pain will be analyzed at each follow-up time point using Mixed-Effects Linear Models to account for repeated measurements and intra-patient correlation. This model includes fixed effects for treatment, time, and the interaction between treatment and time. Random intercepts are used to model individual variability over time. Functional disability scores are assessed via changes in ODI and RMDQ measured at 4, 8, and 12 weeks using Mixed-Effects Linear Models. Both measures are treated as continuous variables, and the analysis accounted for repeated measurements within patients. Mental health assessments will be completed using GAD-7 and PHQ-9 anxiety and depression scores, respectively, using Mixed-Effects Linear Models at 4, 8, and 12 weeks. Serum cortisol levels at 4 weeks will be compared between treatment groups using Student’s t-test for normally distributed data, and the Mann–Whitney U test for non-normally distributed data after assessment of normality.

### Subgroup analysis

Prespecified subgroup analyses will be conducted to explore potential effect modifiers, including injection approach (interlaminar vs. transforaminal), herniation severity (Pfirmann grading system based on lumbar MRI), and patient characteristics such as age, sex, and presence of diabetes. Continuous outcomes (NRS, ODI, RMDQ, GAD-7, PHQ-9) will be analyzed using Mixed-Effects Linear Models with fixed effects for treatment, time, subgroup factor, treatment-by-time, and treatment-by-subgroup interactions, and random intercepts for patients to account for repeated measurements.

### Adverse events

The occurrence of adverse events (AEs) will be reported by treatment group. A Chi-square test or Fisher’s exact test (for categorical data) will be used to compare the occurrence of AEs between the two groups.

### Interpreting instances of missing data

For missing data, we will apply multiple imputation methods to generate plausible values for missing observations under the assumption that data is missing at random. Sensitivity analyses are conducted to assess the impact of missing data by comparing results using different methods for handling missing data (e.g., Last Observation Carried Forward and multiple imputation). All statistical analyses are conducted using SPSS (version 25.0 or higher) or R software. Interim analyses will be used to recalculate the sample size.

### Data and safety monitoring

In this trial, adverse events (AEs) and unintended effects related to the intervention or trial conduct are systematically recorded and managed following a standardized protocol. All solicited AEs are actively monitored, and participants are asked about specific symptoms immediately after the injection, at each scheduled follow-up visit, and via telephone contact during the follow-up period. Any spontaneously reported AEs are documented whenever they occur.

Trained site personnel assess the severity, causality, and duration of each event, and record these details in the case report form. Serious adverse events (SAEs), defined as any unintended medical occurrence resulting in death, being life-threatening, requiring hospitalization or prolongation of hospitalization, or resulting in persistent or significant disability/incapacity, are reported immediately to the principal investigator and the ethics committee.

All AEs, regardless of suspected causality, are included in the final analysis to ensure a comprehensive safety evaluation of dexamethasone palmitate and betamethasone in the treatment of lumbar disc herniation.

A formal independent Data and Safety Monitoring Board (DSMB) was not established in this study due to the routine clinical nature and relatively low-risk profile of epidural steroid injections. However, safety oversight is ensured through continuous monitoring by the principal investigator and periodic review by the institutional ethics committee.

Data management is performed by trained personnel at each participating center. Double data entry is used to minimize transcription errors, with two independent entries for each record. Range and consistency checks are implemented to identify outliers or data entry errors. Regular data audits are conducted to ensure completeness, accuracy, and protocol adherence.

## Discussion

This protocol outlines a multicenter, randomized, double-blind non-inferiority trial comparing dexamethasone palmitate (DEP) with compound betamethasone for epidural injection in patients experiencing lumbar radicular pain. The study is designed to evaluate whether DEP, a novel lipophilic corticosteroid, can achieve similar clinical outcomes to a widely used particulate steroid, while potentially reducing safety concerns and improving pharmacokinetic properties.

Lumbar nerve root compression due to intervertebral disc displacement is a frequent cause of lower limb pain and functional impairment (Deyo and Mirza, 2016). Epidural steroid administration is a common interventional approach, but current evidence does not clarify the optimal choice of corticosteroid (Kreiner et al., 2014; Figueiredo et al., 2020). Particulate agents, such as betamethasone, may offer longer-lasting relief, but carry risks associated with crystal formation.

Dexamethasone palmitate (DEP) is as a prodrug of dexamethasone, consisting of dexamethasone combined with a fat emulsion that includes lecithin (Harasim-Symbor et al., 2016). Compared to dexamethasone, DEP is more readily taken up by activated macrophages due to its fat emulsion properties, which ensures robust distribution in inflammatory tissues while minimizing the risk of adverse effects (Li et al., 2024). Other benefits include a prolonged duration of action due to DEP’s liposome structure. And when mixed with local anesthetics, does not produce significant particulate formation (Hui et al., 2023).

The primary endpoint, leg pain intensity at 4 weeks post-injection, reflects both short-term and early sustained analgesic effects. Secondary endpoints include functional improvement and safety outcomes, including adverse event incidence and serum cortisol levels. A non-inferiority framework was chosen to determine whether DEP can provide outcomes that are clinically comparable to standard particulate therapy, recognizing that particulate agents may have a modest advantage in analgesic duration.

Patient safety remains a priority. Epidural injections carry procedure-specific hazards, such as transient neurological symptoms, inadvertent vascular injection, and infection (Kozlov et al., 2015). All procedures will be conducted under imaging guidance, following standardized techniques. Consent is obtained by trained personnel, who provide detailed information on potential benefits and procedure-related risks. Serious adverse events are promptly reported to investigators and the ethics committee, ensuring close oversight.

Potential sources of variability include the choice of injection route and local anesthetic concentration. Prior research and guideline recommendations suggest these factors minimally affect efficacy, and they will be addressed in the statistical analysis through subgroup evaluation. Multicenter coordination, standardized training, and stratified randomization by baseline characteristics are implemented to maintain consistency across sites.

Several limitations should be acknowledged in this study. First, this trial does not include a comparison with water-soluble dexamethasone, which may be more commonly used in high-risk epidural injections; such a comparison would provide additional clinically relevant information on the relative efficacy and safety of different non-particulate corticosteroids. Second, the study allows variability in injection technique (interlaminar vs. transforaminal) and local anesthetic concentration, reflecting real-world clinical practice where individual physician preferences differ. While this may introduce heterogeneity, it enhances the external validity and generalizability of the findings to routine clinical settings. Third, as a multicenter trial, differences in operator experience, procedural nuances, and patient management across sites may affect consistency, although standardized protocols, training, and stratified randomization by baseline severity are implemented to mitigate these influences. Last, the absence of assessment for pain catastrophizing, such as the Pain Catastrophizing Scale (PCS). Psychological factors like catastrophizing may influence pain perception and treatment response, and their omission may limit the ability to fully account for these potential confounders.

## Conclusion

This study protocol describes a randomized, double-blind, non-inferiority trial designed to evaluate the efficacy and safety of dexamethasone palmitate compared with compound betamethasone for lumbar radiculopathy. By addressing an important gap in evidence regarding the use of dexamethasone palmitate in epidural injection therapy, this study may provide clinically relevant data to guide the selection of corticosteroid formulations in routine practice. The findings are expected to support informed clinical decision-making, optimize therapeutic strategies, and provide a foundation for future studies on alternative steroid options for lumbar radicular pain.
